# Empowering the Invisible: Accelerating Leadership Development for Midcareer Women in Medicine

**DOI:** 10.2196/47801

**Published:** 2023-06-16

**Authors:** Jessica D S Himstedt, Eve Bloomgarden, Purvi Shah

**Affiliations:** 1 University of Illinois at Chicago Chicago, IL United States; 2 NorthShore University Health Systems Chicago, IL United States

**Keywords:** leadership development, women in medicine, gender parity, leadership in medicine, women physicians, midcareer development

## Abstract

Midcareer women physicians face numerous obstacles to career advancement and leadership roles resulting in their contributions and achievements becoming “invisible.” This paper addresses the paradox of increasing professional experience coupled with decreased visibility for women in medicine at this stage in their careers. To address this disparity, the Women in Medicine Leadership Accelerator has developed a leadership skill development program specifically tailored for midcareer women physicians. The program incorporates key principles derived from effective leadership training models and aims to combat systemic barriers while equipping women with the necessary tools to navigate and transform the medical leadership landscape.

Midcareer women physicians often find themselves confronting an array of obstacles to career advancement. These obstacles, which fall on a spectrum including gender biases and inequities, financial implications associated with pregnancy and motherhood, and overt sexual harassment, collectively contribute to their contributions and achievements becoming “invisible.” This contradiction—the rise in professional stature coincident with a decrease in visibility—poses a significant paradox for women at this stage in their careers, a problem well articulated in leadership literature [1]. Both implicit and organizational biases disproportionately affect women's advancement, resulting in an alarming rate of attrition in midcareer women [1-3].

Drawing on the work of Carnes and Bigby [2], it is evident that women in their early career stages receive substantial support and guidance to advance their careers. However, this support appears to dwindle as women progress into midcareer. As women become genuine competitors for limited resources in their fields, they are exposed to the pervasive culture of inequities and systemic bias in academic medicine [2].

Within the Women in Medicine Leadership Accelerator, we have created a unique leadership skill development program designed explicitly for midcareer women physicians. This program is rooted in key principles derived from effective leadership training models, which aim to combat systemic barriers and equip women with the necessary tools to navigate and ultimately transform the medical leadership landscape. A recent meta-analysis identified four key elements that positively impacted results from training reactions to organizational outcomes [4]. These were:

1. Active learning strategies: The most effective programs encouraged participants’ active involvement, providing them with opportunities to apply what they have learned in real or simulated situations.
2. Feedback and coaching: Participants showed significant improvement when they received regular feedback on their progress and coaching to help them refine their leadership skills and behaviors.
3. Program duration: Longer programs that provided opportunities for practice and reinforcement were more effective than shorter ones. This is reflected in our program’s design, which spans a considerable 6-month period, allowing participants to learn, practice, and reinforce their leadership skills.
4. Consideration of participant characteristics: The effectiveness of leadership programs varied based on participant characteristics such as age and prior leadership experience. This underscores the importance of tailoring programs to meet the specific needs and context of the participants.

Informed by these insights, our leadership accelerator program is designed to provide a robust and tailored learning experience. Participants are given opportunities to actively learn, practice leadership skills, and receive feedback within the group; they also benefit from personalized one-on-one coaching. Our program aims to amplify the visibility and leadership potential of midcareer women in medicine through this comprehensive approach.

A critical tool that has not been widely implemented in midcareer development is 360° feedback. This assessment facilitates feedback collection from different perspectives about a leader’s effectiveness across specific competencies. This tool aims to promote program participants’ self-awareness and guide their understanding of key strengths and growth areas [4], and is a cornerstone of our program.

There are major benefits to the use of 360° feedback. The synthesis of responses can guide targeted development activities, including training, coaching, and mentoring programs [5]. One of the most significant benefits of 360° feedback programs is increased self-awareness among leaders. It provides a comprehensive understanding of their skills, behaviors, and performance from the perspective of various people in the organization, including peers, direct reports, and supervisors. This awareness can lead to improved leadership effectiveness [6].

With two cohorts having completed the leadership accelerator training, we are able to measure its effectiveness through participant satisfaction and self-report. Additionally, 360° pre- and postprogram surveys provide an effective measure of behavioral change, and we are also assessing early organizational impacts through objective evaluations of promotions, publications, grants received, and other productivity metrics where gender parity is crucial.

Anecdotes from participants underline the potential of our initiative in bridging the leadership gap for midcareer women in medicine. Participants credit the program for significant professional accomplishments, including delivering keynote speeches at conferences, journal publications, securing grants, and earning promotions. Many of these accomplishments are highlighted below, in the participants’ own words.

One participant remarked:

After joining the program, I have gained the confidence to take on more responsibilities. I was invited to give a keynote speech at a prestigious conference, and recently, my article was published in a renowned journal.

Another shared her experience, saying:

The program has been transformational for me. I have been able to secure grants for my public policy work, earn a promotion, and even find the courage to take on roles that I would have hesitated to pursue earlier. I now feel more equipped to handle the challenges of my profession.

Another cohort member stated:

I feel more comfortable as an expert. I am giving three talks at national conferences and had a piece published about the work at my organization in a well-read publication. My boss is advocating for me to take on a larger role within my organization.

Similarly, a participant who transitioned to a new role at a new institution submitted multiple awards and successfully relocated her family across the country credited the program for her success reports:

I couldn't have navigated through all these changes smoothly without the skills and insights I gained from the program.

Another woman, after completing our program, took up a directorship with a clearly defined mission and vision. She said:

I felt prepared and ready for the challenges ahead. I also published multiple papers, including one focused on gender equity and leadership. I have set better boundaries at work and home and feel in control of my professional life.

Initiatives such as our leadership accelerator program signify not only a beacon of hope but a model for success, offering vital support, feedback, camaraderie, and enhanced visibility to midcareer women physicians. Through this, we envisage enabling these accomplished professionals to not only persist on their chosen trajectories but also rise to leadership roles that they aspire toward and are more competently prepared for by equipping them with the support that is lacking at this stage in their careers. Such efforts could represent a transformative shift in medicine, ushering in a more inclusive and equitable landscape for all.
